# Targeting the BCL-2-regulated apoptotic pathway for the treatment of solid cancers

**DOI:** 10.1042/BST20210750

**Published:** 2021-09-28

**Authors:** W. Douglas Fairlie, Erinna F. Lee

**Affiliations:** 1 Cell Death and Survival Laboratory, Olivia Newton-John Cancer Research Institute, Heidelberg, Victoria 3084, Australia; 2 Cell Death and Survival Laboratory, School of Cancer Medicine, La Trobe University, Bundoora, Victoria 3086, Australia; 3 La Trobe Institute for Molecular Science, La Trobe University, Melbourne, Victoria 3086, Australia

**Keywords:** apoptosis, BCL-2, BH3-mimetics, cancer

## Abstract

The deregulation of apoptosis is a key contributor to tumourigenesis as it can lead to the unwanted survival of rogue cells. Drugs known as the BH3-mimetics targeting the pro-survival members of the BCL-2 protein family to induce apoptosis in cancer cells have achieved clinical success for the treatment of haematological malignancies. However, despite our increasing knowledge of the pro-survival factors mediating the unwanted survival of solid tumour cells, and our growing BH3-mimetics armamentarium, the application of BH3-mimetic therapy in solid cancers has not reached its full potential. This is mainly attributed to the need to identify clinically safe, yet effective, combination strategies to target the multiple pro-survival proteins that typically mediate the survival of solid tumours. In this review, we discuss current and exciting new developments in the field that has the potential to unleash the full power of BH3-mimetic therapy to treat currently recalcitrant solid malignancies.

## Introduction

**Figure 1.**
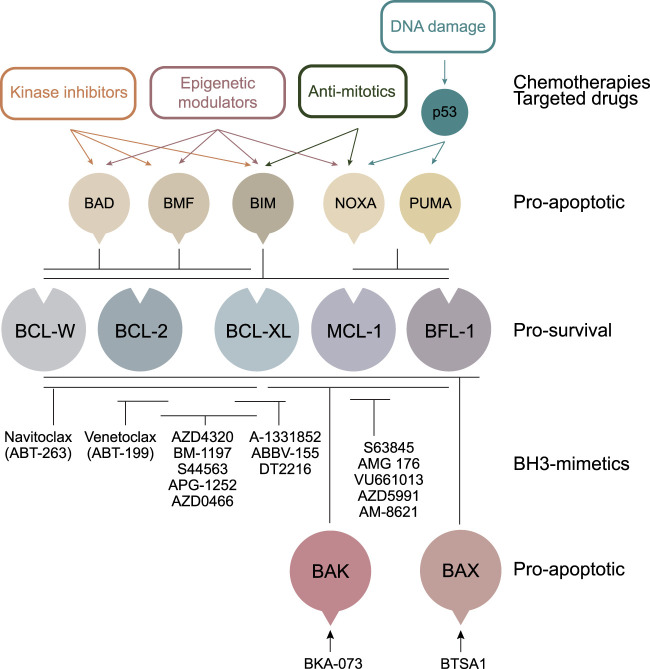
Anti-cancer drugs often work by the induction of apoptosis. Many anti-cancer drugs exert their cytotoxic effects by indirectly up-regulating the pro-apoptotic BH3-only proteins or by decreasing the expression of the pro-survival BCL-2 proteins. In contrast, BH3-mimetics directly antagonise the pro-survival BCL-2 proteins, bypassing defects in upstream signalling pathways such as those mediated by p53, which is commonly inactivated in cancers. Direct activators of BAX or BAK have also been developed as an alternate mechanism for inducing apoptosis.

Drugs known as the ‘BH3-mimetics’ trigger cell death by antagonising the action of the pro-survival members of the BCL-2 protein family (Figure 1). These proteins, which include BCL-2, BCL-XL, MCL-1, BCL-W and BFL-1, are key negative regulators of the intrinsic apoptosis pathway which is deregulated in most, if not all cancers. Pro-survival BCL-2 proteins also contribute to resistance to standard treatments such as chemo- and radiotherapies that act through cell death induction (Figure 1). The BH3-mimetics are so-called because they mimic the natural ligands for pro-survival proteins — the pro-apoptotic BH3-only proteins. As with BH3-only proteins, binding of BH3-mimetics to their pro-survival targets leads to apoptosis induction via two mechanisms: (1) the displacement of prebound pro-apoptotic BH3-only proteins to activate BAX or BAK, and (2) through the release of activated BAX or BAK themselves [1,2]. This latter mechanism is sufficient for apoptosis induction by BH3-mimetics as the absence of BH3-only proteins in cells does not negate their killing activity [2,3]. The discovery of how these ligands bind their targets in the late 1990s inspired the development of their small molecule counterparts. However, it was not until 2016 that the first of these drugs, Venetoclax which specifically targets BCL-2, was granted FDA approval for relapsed chronic lymphocytic leukaemia (CLL) patients with 17p deletion.

Venetoclax has now been approved as standard-of-care treatment for CLL in combination with Rituximab or Obinutuzumab depending on the treatment status of the patient [4,5], with chemotherapies in acute myeloid leukaemia (AML) [6], and is undergoing >230 clinical trials in a variety of haematological cancers. However, the clinical use of Venetoclax and other BH3-mimetics in solid cancer treatment is not nearly as well-advanced. In this review, we primarily discuss the contributions of BCL-2 pro-survival proteins in solid cancers and the challenges that remain in the application of BH3-mimetics for their treatment.

## Pro-survival protein dependencies in solid cancers: single-agent BH3-mimetic applications

### BCL-XL

Prior to Venetoclax, Navitoclax was the first BH3-mimetic to enter clinical trials, binding with high affinity to BCL-2, BCL-XL and weaker to BCL-W [7]. In pre-clinical studies, Navitoclax monotherapy elicited durable tumour regression in small cell lung cancer (SCLC) comparable to clinically approved cytotoxic agents [7–9], and enhanced the *in vivo* activity of multiple therapeutic agents, including anti-mitotics and DNA-damaging agents in solid cancers including non-small cell lung (NSCLC), breast, mesothelioma and ovarian cancer [8,10–13]. Other BCL-XL and BCL-2 dual inhibitors developed subsequently (e.g. AZD4320, BM-1197, S44563, APG-1252) also demonstrated similar long-lasting tumour regression in SCLC, gastric and uveal melanoma xenograft models [14–16].

Further studies demonstrated that responses to Navitoclax (or its predecessor ABT-737 with an identical pro-survival binding profile) in solid cancers was mainly due to inhibition of BCL-XL and not BCL-2 [11]. This is consistent with the amplification of the chromosomal region, encompassing the *BCLX* gene, found in many solid tumours [17]. Further studies have now validated BCL-XL as the critical survival factor for a range of solid cancers including colorectal, bladder, pancreatic and cervical cancers [18–21].

Due to the early promise seen with targeting BCL-XL in solid cancers, a programme to develop BCL-XL-selective inhibitors was initiated. The first orally bioavailable BCL-XL-selective inhibitor A-1331852 demonstrated modest single-agent activity in NSCLC, mesothelioma, breast, ovarian and colorectal cancer xenograft models [8,11,22]. However, combinations with chemotherapies (e.g. docetaxel or irinotecan) led to significant increases in the amplitude and durability of responses [11,22].

Whilst the pre-clinical data supporting BCL-XL targeting in solid cancers is compelling, its inhibition also causes significant thrombocytopaenia due to the dependence of platelets on BCL-XL for their survival [23]. This dose-limiting toxicity resulted in several trials with Navitoclax being halted and probably underlines why A-1331852 has yet to enter the clinic. It also fuelled the development of BCL-2-selective inhibitors like Venetoclax for use in cancers reliant on BCL-2, circumventing the thrombocytopaenia arising from BCL-XL inhibition [24].

### MCL-1

Like *BCL-XL*, focal amplification of the *MCL1-*containing chromosomal region occurs in ∼11% of solid cancers [17] including NSCLC [25–27] and melanoma [28]. Gene knockdown studies proved that amplified MCL-1 enabled the survival of these tumours [17,27,29]. MCL-1 is also highly expressed in most breast cancer subtypes, for example, triple-negative and HER2-amplified breast cancers [30–32], enabling tumour cell survival and chemoresistance. Its overexpression also correlates with high tumour grade and poor patient survival [33,34].

Several MCL-1 antagonists have now been successfully developed [35,36] with S63845 being the first applicable for *in vivo* use [37]. This was followed by several others including AMG 176, VU661013, AZD5991 and AM-8621 [38–41]. As with single-agent BCL-XL inhibition, MCL-1 inhibitors used as monotherapy are only marginally effective in solid tumour-derived cell lines [37] such as patient-derived xenograft (PDX) models of breast cancer [31] and *KRAS*-mutant NSCLC [40]. However, co-treatment of S63845 with oncogenic kinase inhibitors (e.g. Lapatinib, Tarceva, Trametinib) or other chemotherapeutic agents (e.g. docetaxel, trastuzumab) enhances cytotoxic responses [31,37,40], emphasising the need for prioritisation of BH3-mimetic combination therapies in the clinic, which will be discussed further below.

### BCL-2

Although usually associated with haematological cancers, amplification of the *BCL2* locus on chromosome 18q21 leads to BCL-2 overexpression and occurs in up to 80% of SCLC [42,43]. In pre-clinical studies, Navitoclax/ABT-737 demonstrated activity in SCLC cell lines and xenograft models [9,44] warranting entry into Phase I/II clinical trials. However, outcomes were disappointing [45,46] and this failure was attributed to the dose-limiting thrombocytopaenia from BCL-XL co-inhibition, restraining the magnitude of BCL-2 inhibition achievable. Accordingly, Venetoclax was investigated for SCLC treatment [47] as BCL-2 expression was a predictive biomarker for Venetoclax responsiveness. Venetoclax, in combination with chemotherapeutic agents, entered clinical trials for SCLC (NCT04422210, NCT04543916) but these studies were halted citing strategic prioritisation and broader development.

In addition to SCLC, ∼20% of *MYCN*-amplified neuroblastoma cells are highly sensitive to Venetoclax [48]. BCL-2 is expressed significantly higher in neuroblastoma cells compared with other solid cancer lines, whilst BCL-XL expression is reduced, consistent with their responsiveness to Venetoclax *and* Navitoclax when *MYCN* is amplified [48–51]. In breast cancer, BCL-2 is an estrogen-responsive gene overexpressed in ∼85% of estrogen receptor (ER)-positive breast cancers [52]. Therefore, when Venetoclax was combined with endocrine therapy central to the management of ER-positive breast cancer, marked improvements in tumour response in ER-positive PDX models were observed [53]. These findings validated BCL-2 as a therapeutic target for the treatment of ER-positive breast cancer and led to the very first clinical study with Venetoclax for a solid cancer. Critically, the combination of Veneoclax with endocrine therapy had a tolerable safety profile with notable activity in ER- and BCL-2-positive metastatic breast cancer in a Phase Ib study [54].

Whilst BCL-2 targeting has achieved remarkable clinical success in haematological malignancies, these results demonstrate the potential of targeting this eponymous member of the BCL-2 family for the treatment of solid cancers.

### BCL-W and BFL-1

Whilst BCL-XL, MCL-1 and BCL-2 have received most of the attention in oncology drug development, the lesser discussed pro-survival BCL-2 proteins, BCL-W and BFL-1, also have important roles in cancer. Elevated levels of BFL-1 have been primarily described in blood cancers [55,56]. In solid tumours, aberrant BFL-1 expression has been documented in the stomach [57] and breast cancers [58,59] as well as malignant melanoma, though its requirement for melanoma cell survival varies between studies [60–66]. As with BFL-1, BCL-W is highly expressed in human B-cell lymphomas [67,68]. In solid malignancies, deregulation of BCL-W expression has been reported in bladder cancer, colorectal carcinoma and lung cancer [69–71].

Importantly, BCL-W and BFL-1 are intrinsic resistance factors to BH3-mimetics targeting BCL-XL, MCL-1 and BCL-2 [72–76]. Currently, there are no known BFL-1- or BCL-W-specific inhibitors in development despite programmes that initially set out to achieve this [77,78]. In the absence of such direct inhibitors, molecules that indirectly modulate their levels could potentially be applied instead [73].

## Targeting multiple pro-survival proteins with BH3-mimetics for solid cancer treatment

Although rare, acute sensitivity to single-agent BH3-mimetics does occur, especially in haematological cancers and some solid cancers, as discussed above. However, most solid tumours are dependent on more than one pro-survival protein which dictates BH3-mimetic efficacy. These co-dependencies are discussed below.

### BCL-XL and MCL-1 co-targeting

Systematic studies using BH3-mimetic ‘parsing’ defined the pro-survival protein dependency of a large panel of cancer cell lines from ten tissues of origin and showed a striking co-dependency of ∼90% of these on BCL-XL and MCL-1 [20]. Notably, targeting this specific combination led to synergistic activity in ∼50% of solid tumour cell lines representing melanoma, breast, colorectal, brain, ovarian and pancreatic cancers. Multiple solid tumour-specific studies have similarly highlighted the effectiveness of co-targeting BCL-XL and MCL-1. In squamous cell lung carcinoma, dual inhibition of MCL-1 and BCL-XL induced synergistic tumour cell death, and when combined with fibroblast growth factor receptor (FGFR)-targeted therapy, produced durable treatment responses in *FGFR1-*overexpressing lung squamous cell carcinoma [79]. Similar results were also observed in malignant pleural mesothelioma, NSCLC and colorectal cancer [8,12,19,40,80,81]. In these cancers, BCL-XL is the dominant survival factor as its sole targeting had a greater effect compared with the targeting of MCL-1 alone. In breast cancer, BCL-XL serves as a resistance factor to MCL-1-specific inhibitors, further exemplifying the need to target both these pro-survival proteins for effective killing [82–84]. In malignant melanoma, prostate, and *KRAS*-mutant NSCLC, no one pro-survival protein appeared to govern their survival as targeting both MCL-1 and BCL-XL were highly efficacious [40,62,85]. Gene set enrichment studies have suggested that the shifting dependence of solid tumours on both BCL-XL and MCL-1 or just BCL-XL alone is associated with epithelial-mesenchymal transition (EMT) [20] where both proteins are required for survival of epithelial cells, but mesenchymal cells become BCL-XL dependent due to induction of NOXA, an MCL-1 antagonist.

### BCL-2 and MCL-1 co-targeting

Whilst BCL-XL and MCL-1 co-targeting with BH3-mimetics appears to be the ‘magic formula’ for delivering the *coup de* grâce to most solid cancers, the inhibition of BCL-2 and MCL-1 with BH3-mimetics has also been shown to be effective in some cases. For example, in *MYCN*-amplified neuroblastoma detailed previously, MCL-1 mediates resistance to Venetoclax, where sensitivity depends on *MYCN*-regulated NOXA expression to antagonise MCL-1 [48,49,51]. Accordingly, agents that down-regulate MCL-1 (e.g. Aurora kinase A inhibitor MLN8237, Cyclophosphamide, MYC activators) sensitise *MYCN*-amplified neuroblastoma cells to Venetoclax, providing durable responses in xenograft models [48,51]. These promising pre-clinical results have now led to Phase I clinical trials in this highly resistant cancer (NCT03236857, [86]).

Whilst BCL-XL and MCL-1 antagonism in melanoma leads to synergistic killing, co-inhibition of MCL-1 and BCL-2 is also effective *in vitro*, albeit to a lesser extent [62], and in *in vivo* models (e.g with Venetoclax and the clinically applicable MCL-1 inhibitor S64315) [64]. Notably, BCL-2 expression in patients with BRAF^wild-type^ melanomas, for which there is little in the way of targeted therapies, is higher compared with BRAF^mutant^ melanomas [87].

## Circumventing toxicities associated with BH3-mimetic combinations

The use of BH3-mimetic combinations to target multiple pro-survival proteins appears to be an obvious means to combating solid cancers. However, given the key and often compensatory roles these proteins play in non-malignant cells, co-inhibition of two or more pro-survival proteins potentiates the opportunity for associated toxicities. The simultaneous disarmament of BCL-XL and MCL-1 with BH3-mimetics in mice is lethal due to acute liver toxicity [12,79] as both these proteins are critical for hepatocyte survival [88]. These pro-survival proteins are also essential for megakaryocyte survival [89]. Likewise, the loss of a single allele each of *BCL2* *and MCL1* in mice leads to reduced organismal size due to a reduction in body cellularity [90]. Nevertheless, co-targeting BCL-2 (with Venetoclax) and MCL-1 (with S64315 or AMG176) is in dose-finding clinical studies for the treatment of haematological malignancies (NCT03672695, NCT03797261) [91]. However, the progression of this combination will rely on the evaluation of the cardiac toxicity signal that arose in Phase I trials with AMG176/AMG397 (NCT03465540, NCT02675452).

One avenue to circumvent these toxicities is to combine BH3-mimetics with agents that indirectly modulate the expression of BCL-2 family members in a tumour-specific manner — either by up-regulating pro-apoptotic (Figure 1) or down-regulating pro-survival proteins. The role of BCL-2 proteins as mediators of the cytotoxic response to chemotherapeutic agents is well-established [92]. Given the limited efficacy of BH3-mimetic monotherapy in solid tumours, the potential of combining BH3-mimetics with standard-of-care therapeutics has already been demonstrated as described above, and multiple clinical trials of BH3-mimetics combined with anti-cancer agents are in progress for the treatment of solid malignancies (Table 1).

**Table 1.** Some of the clinical trials conducted with BH3-mimetics in solid cancers

Disease	BH3-mimetic	Target	Chemotherapeutic agent	Clinical study	Status	Relevant references
ER+ breast cancer	Venetoclax	BCL-2	Tamoxifen	ISRCTN98335443	Closed	[53,54,155]
ER + HER2− breast cancer	Venetoclax	BCL-2	Fulvestrant	NCT03584009	Completed	[156]
HER2+ breast cancer	Venetoclax	BCL-2	Trastuzumab Emtansine	NCT04298918, CO41863	Terminated, Closed	
Neuroblastoma	Venetoclax	BCL-2	Cyclophosphamide	NCT03236857, M13-833	Recruiting	[49,51,86]
SCLC	Venetoclax	BCL-2	Atezolizumab, Carboplatin, Etoposide	NCT04422210	Terminated	[47]
SCLC	Venetoclax	BCL-2	Irinotecan	NCT04543916	Withdrawn	[157]
SCLC	Venetoclax	BCL-2	ABBV-075	NCT02391480	Completed	[137,139]
SCLC, solid cancers	APG-1252	BCL-XL, BCL-2, BCL-W	—	NCT03387332	Recruiting	[16,158]
Solid cancers	Navitoclax	BCL-XL, BCL-2, BCL-W	Trametinib	NCT02079740	Recruiting	[114,159]
Solid cancers	Navitoclax	BCL-XL, BCL-2, BCL-W	Gemcitabine	NCT00887757	Completed	[160]
Solid cancers	Navitoclax	BCL-XL, BCL-2, BCL-W	Docetaxel	NCT00888108	Completed	[161]
Solid cancers	Navitoclax	BCL-XL, BCL-2, BCL-W	Etoposide, Cisplatin	NCT008878449	Completed	[162]
Solid cancers	Navitoclax	BCL-XL, BCL-2, BCL-W	Sorafenib	NCT02143401	Active, not recruiting	[163]
Melanoma, solid cancers	Navitoclax	BCL-XL, BCL-2, BCL-W	Dabrafenib, Trametinib	NCT01989585	Recruiting	[164]
Solid cancers	AZD0466	BCL-XL, BCL-2	—	NCT04214093	Completed	[147]
Solid cancers	ABBV-155	BCL-XL	Taxanes	NCT03595059	Recruiting	[149]
Solid cancers	DT2216	BCL-XL	—	NCT04886622	Not yet recruiting	[150]

### BH3-mimetics and anti-mitotic agents

Systematic analysis of Navitoclax's capacity to enhance the activity of clinically relevant agents across a spectrum of solid tumours revealed the greatest synergy with anti-mitotic drugs such as Docetaxel and Paclitaxel [10]). This combination significantly improves responses in a range of solid cancers including NSCLC, ovarian, gastric and breast cancer [10,13,93,94]. As mentioned above, the Aurora Kinase A inhibitor MLN8237 that also induces mitotic arrest, enhances Venetoclax activity in *MYCN*-amplified neuroblastoma [48] and of Navitoclax in pancreatic adenocarcinoma [95].

The synergy seen with anti-mitotic agents is attributed to MCL-1 phosphorylation and its proteasomal degradation following mitotic arrest [96,97]. Anti-tubulin chemotherapeutics also result in BCL-XL phosphorylation causing its functional inactivation [98,99], or the up-regulation of pro-apoptotic BIM [100,101] or NOXA [48,102]. The consequence of all these is the lowering of the apoptotic threshold, sensitising cells to direct BCL-XL or BCL-2 antagonism.

### BH3-mimetics and oncogenic kinases

A major class of drugs under investigation for use in combination with BH3-mimetics are oncogenic kinase inhibitors. These include kinases along the mitogen-activated protein kinase (MAPK) pathway which serves as a critical bridge between extracellular signals and intracellular responses, or the cyclin-dependent kinases which co-ordinate cellular events including cell proliferation and survival (e.g. CDK9). Critically, such kinases regulate BCL-2 proteins to favour cellular survival. This occurs by the up-regulation/stabilisation of pro-survival proteins such as MCL-1 [103–106] or through post-translational modifications such as phosphorylation leading to functional inactivation or proteasomal degradation of pro-apoptotic proteins such as BIM [107,108], BAD [109,110] or BMF [111]. Notably, the predominant response to oncogenic kinase inhibition is cytostatic as opposed to cytotoxic as the elevated pro-survival protein levels in cancer cells buffer any induced pro-apoptotic BH3-only proteins [112]. Hence, BH3-mimetics can lower the apoptotic threshold to unleash the pro-apoptotic power of oncogenic kinase inhibitors.

Cancer-associated alterations of MAPK signalling arise from activating mutations to any of the downstream effector molecules along the pathway (e.g. RAS/RAF/MEK/ERK that converge on the PI3K/AKT pathway) or to upstream tyrosine kinase receptors (e.g. EGFR). As most solid cancers possess mutations along this signalling node [113] they are attractive targets to investigate in combination with BH3-mimetics. Such combinations result in potent efficacy in solid cancer models including NSCLC [10,40,114–117], melanoma [37,112,118], prostate [37,85] and colorectal cancers [112]. In almost all examples, kinase inhibition up-regulates pro-apoptotic BH3-only proteins such as BIM, PUMA or BMF [85,111,112,114–117,119]. In some cases, it is associated with decreased MCL-1 expression or its increased degradation [10,85]. Notably, these combinations of BH3-mimetics are well-tolerated and in clinical trials in solid tumours (NCT02520778, NCT02143401, NCT02079740, NCT01989585).

Another class of kinases being explored for use in combination with BH3-mimetics are cyclin-dependent kinase inhibitors, specifically those involved in gene transcription such as CDK9. CDK9 regulates transcription of oncogenic genes including MYC and is essential for the maintenance, growth and chemoresistance of many solid cancers including breast [120], lung [121], osteosarcoma [122], pancreatic [123] and melanoma [124] and is prognostic of worse overall and disease-free survival [122,123]. In all cases, CDK9 inhibition leads to the suppression of tumour formation and induces apoptosis via reduction of MCL-1 expression [84,125-128]. Therefore, CDK9 inhibitors offer an alternative approach to target MCL-1 activity indirectly in combination with BH3-mimetics [125,127,128]. Accordingly, the combination of Venetoclax and CDK9 inhibitors (e.g. Voruciclib, A-1592668, AZD4573, LS-007) [128–131] provides superior efficacy over monotherapy in haematological malignancies, and is well-tolerated *in vivo*. In solid cancers, the combination of Dinaciclib (which targets CDKs 1, 2, 5, 9) with A-1331852 or ABT-737 to target BCL-XL showed promising responses in soft-tissue sarcoma cells [132]. However, *in vivo* this combination led to liver toxicity linked to hepatocyte apoptosis, likely due to the broad action of Dinaciclib against CDKs in multiple cellular processes, hence specific CDK9 inhibitors will likely have more promise.

Based on pre-clinical studies, the scope for strategies based on BH3-mimetics in combination with oncogenic kinases is vast and promising. Whilst only two classes of oncogenic kinases have been described in detail here, other kinase families that contribute to the aetiology of solid tumours including the PI3K/Akt/mTOR pathway are also being explored in combination with BH3-mimetics [133].

### BH3-mimetics and epigenetic modulators

Another promising approach being explored in combination with BH3-mimetics in solid cancers is the inhibition of epigenetic modulators such as bromodomain and extra-terminal (BET) family proteins or histone deacetylases (HDAC). Originally demonstrated in haematological malignancies, epigenetic modulators such as the HDAC inhibitor Vorinostat in combination with ABT-263/ABT-737 facilitated synergistic tumour cell apoptosis. Similarly, in solid cancers including rhabdomyosarcoma, breast cancer, SCLC and melanoma, synergistic killing was achieved, either through transcriptional up-regulation of pro-apoptotic BIM, NOXA, PUMA or BMF [134–139] or suppression of BCL-2 and/or BCL-XL expression [137,138,140,141]. In melanoma, the BET inhibitor JQ1 suppressed BFL-1 expression through inhibition of its transcriptional regulator, NF-kB [138]. As BFL-1 remains untargeted by BH3-mimetics, this effect of BET inhibition could be a strategy to circumvent BFL-1 dependency of tumours such as melanoma [60,63,66].

Intriguingly, treatment with JQ1 can also lead to an increased tumour cell dependence on pro-survival proteins resulting in resistance to pharmacological modulators of epigenetic regulation [142–144]. For example, in triple-negative breast cancer, gain of a superenhancer was detected at the *BCLX* locus and served as a mechanism of maintaining BCL-XL expression and resistance to JQ1 [143]. Accordingly, BH3-mimetics could serve to target overexpressed pro-survival protein(s) or redistribute any pro-apoptotic BH3-only proteins they sequester. Encouragingly, the synergistic killing afforded by combinations of BH3-mimetics and epigenetic regulators appears confined to cancer cells [134,138].

### Other combinations

In addition to the combinations of BH3-mimetics with the targeted therapies discussed above, combinations with less specific DNA-damaging chemotherapies have also been explored in solid cancers. Like the drugs described above, such agents can enhance the anti-tumour activity of BH3-mimetics in multiple solid cancers including ovarian cancer [10,94], mesothelioma [8] and sarcomas [145,146] where chemotherapy is often the mainstay treatment, though the mechanism(s)-of-action for the enhanced killing is not well explored. However, it would be reasonable to assume that this is likely mediated by up-regulation of BH3-only proteins such as BIM or the p53-responsive genes PUMA and NOXA, following DNA damage [92].

## The future of BH3-mimetic therapy for solid cancers

Clinical susceptibility to single-agent BH3-mimetic therapy has been mostly limited to haematological cancers. To broaden the range of cancers that can be tackled with BH3-mimetics, strategies that safely achieve the antagonism of multiple pro-survival proteins must be considered. Whilst combination therapies with agents that preferentially impact cancer cells is being investigated, another exciting approach being explored are technologies that enable direct delivery of BH3-mimetics to tumour cells.

A Phase I global clinical trial to evaluate the safety and tolerability of the dendrimer-based nanoparticle formulation of a dual BCL-2/BCL-XL inhibitor, AZD0466, in haematological and solid cancers is currently underway (NCT04214093) [147]. No combination trials of AZD0466 with either ‘unconjugated’ BH3-mimetics targeting for example MCL-1, or chemotherapeutic agents have yet been announced. However, pre-clinical studies investigating the co-administration of AZD0466 *and* Cisplatin in a mesothelioma xenograft model demonstrate improved tumour killing with minimal thrombocytopaenia associated with BCL-XL targeting [80]. Another approach that directs BH3-mimetics to tumour cells is to conjugate them with antibodies targeting unique or overexpressed antigens preferentially found on cancer cells. One such antigen is B7-H3, an immune regulator protein widely expressed by solid tumours including melanoma and NSCLC [148]. The compound ABBV-155, which is a first-in-class antibody drug-conjugate comprising a BCL-XL inhibitor conjugated to an anti-B7H3 antibody, is now in clinical trials either as monotherapy or in combination with taxanes for the treatment of relapsed/refractory solid tumours including NSCLC, SCLC and breast cancer (NCT03595059). The results to date demonstrate a tolerable safety profile and anti-tumour activity [149].

In addition to strategies exploiting antigens or microenvironmental properties unique to cancer cells, another approach to reduce toxicities is to apply proteolysis-targeting chimeras (PROTACs) technology to BH3-mimetics. For example, by targeting BCL-XL to the Von Hippel-Lindau E3 ligase, which is minimally expressed in platelets, the PROTAC DT2216 resulted in reduced thrombocytopaenia and enhanced activity with chemotherapeutics in solid cancer xenograft models of SCLC, triple-negative breast cancer, prostate, colon and liver cancers compared with Navitoclax [150]. Importantly, these combinations were well-tolerated and DT2216 is now in clinical trials (NCT04886622) for use in relapsed/refractory malignancies including solid tumours.

The activation of BAX and/or BAK is the primary outcome essential to the killing activity of BH3-mimetics therapy. Whilst not a focus of this review, it would be remiss of us not to highlight the active investigation into the development of compounds to directly activate BAX or BAK [151–154]. For example, a small molecule compound, BTSA1, was developed to directly activate BAX and, promisingly, demonstrated selective killing of AML cells whilst sparing normal cells [154]. Likewise, a small molecule BAK activator, BKA-073, was shown to be effective against SCLC and NSCLC *in vivo*. Furthermore, consistent with the overexpression of BCL-2 in SCLC potentially blunting an apoptotic response, synergistic killing was observed when BKA-073 was used in combination with Venetoclax [152]. Therefore, whilst still in proof-of-concept development, this promising approach to induce apoptosis in cancer cells warrants further investigation.

Decades of research have yielded clinically applicable drugs targeting the pro-survival members of the BCL-2 family. Whilst the path to achieving killing efficacy is now clear, how we achieve this remains the challenge in the application of BH3-mimetic therapy for the future treatment of solid cancers. The combination strategies discussed above offer significant promise, so long as a safe therapeutic window or ‘sweet spot’ can be identified.

## Perspectives

- BH3-mimetics drugs that directly antagonise the pro-survival proteins of the BCL-2 family to induce apoptosis are clinically approved for the treatment of some haematological cancers. However, the use of BH3-mimetics for the treatment of solid cancers is less established.
- Solid cancers are often reliant on multiple pro-survival proteins for their survival. Whilst simultaneous co-targeting of these survival factors with BH3-mimetics induces efficient killing, it can also lead to adverse effects on normal cells.
- As such, combination strategies utilising BH3-mimetics with other anti-cancer agents that indirectly modulate the pro-survival function of BCL-2 proteins, are being investigated in solid cancers as an alternate and safer approach. In addition, tumour-directed BH3-mimetics are being developed to circumvent the issues arising from the targeting of normal cells.

## Open Access

Open access for this article was enabled by the participation of La Trobe University in an all-inclusive *Read & Publish* pilot with Portland Press and the Biochemical Society under a transformative agreement with CAUL.

## Abbreviations

AML: acute myeloid leukaemia
BAD: BCL2 associated agonist of cell death
BCL-2: b-cell lymphoma 2
BCL-W: b-cell lymphoma w (WEHI)
BCL-XL: b-cell lymphoma-extra large
BET: bromodomain and extra-terminal
BFL-1: bcl-2-related gene in foetal liver 1
BH3: bcl-2 homology 3
BIM: bcl-2-like protein 11
BMF: bcl-2 modifying factor
CDK: cyclin-dependent kinase
CLL: chronic lymphocytic leukaemia
EGFR: epidermal growth factor receptor
FGFR1: fibroblast growth factor receptor 1
HDAC: histone deacetylase
MCL-1: myeloid cell leukaemia 1
mTOR: mechanistic target of rapamycin
MYCN: v-myc myelocytomatosis viral related oncogene, neuroblastoma derived
NF-κB: nuclear factor kappa-light-chain-enhancer of activated B cells
NOXA: phorbol-12-myristate-13-acetate-induced protein 1 (latin for damage)
NSCLC: non-small cell lung cancer
PDX: patient-derived xenograft
PI3K: phosphatidylinositol-3-kinase
PROTAC: proteolysis-targeting chimaera
PUMA: p53 up-regulated modulator of apoptosis
SCLC: small cell lung cancer
